# Inhibitory action of three lactic acid bacteria cultures on some food-borne pathogens during pickling of green olive fruits

**DOI:** 10.1016/j.heliyon.2022.e11693

**Published:** 2022-11-18

**Authors:** Abdul-Raouf Almohammadi, Seham Abdel-Shafi, Eman Tartour, Gamal Enan

**Affiliations:** a Department of Botany and Microbiology, Faculty of Science, Zagazig University, 44511, Zagazig, Egypt; b Department of Sciences, King Khalid Military Academy, P.O. Box 22140, Riyadh, 11495, Saudi Arabia

**Keywords:** Lactic acid bacteria (LAB), Food-borne pathogens, Green olive pickling, Transmission electron microscopy (TEM)

## Abstract

The healthy olive pickles are those ones which are made by using lactic acid bacteria (LAB) as starter and protective cultures. Three lactic acid bacteria (LAB) strains namely: *Lactobacillus plantarum* LPS10 (*L. plantarum*), *Lactobacillus fermentum* PP17 (*L. fermentum*) and *Pediococcus acidilactic*i MH512904 (*P. acidilactici*) were used for inhibition of some food-borne bacterial pathogens such as *Listeria monocytogenes* LMG10470 (*L. monocytogenes*), *Staphylococcus aureus* ATCC25923 (*S. aureus*), *Bacillus cereus* ATCC14579 (*B. cereus*) and *Escherichia coli* ATCC25922 (*E. coli*) in Brain Heart Infusion Broth (BHIB) and during pickling of green olive fruits. Cell free supernatants (CFS) from LAB showed distinctive inhibition of the indicator bacterial pathogens used and the inhibitory activity was more pronounced against Gram positive bacteria than that found against Gram negative *E. coli* strain used; the inhibitory activity of CFS was more pronounced than that obtained by neutralized cell free supernatants (NCFS). Cultures of LAB were used for inhibition of the food-borne pathogens during pickling of green olive fruits. The food-borne bacterial cells grew in olive pickles brine (control) and their growth (CFU/mL) decreased in treated pickles samples and distinctive differences in growth values (CFU/mL) were observed between control and treated samples. CFS from *L. plantarum* affected target cells of both *L. monocytogenes* and *E. coli* and caused cell deformations, cell shrinkage and cell lysis as showed by Transmission Electron Microscopic (TEM) examinations.

## 1 Introduction

Pickling is an ancient way for food preservation in salted medium; vegetables were found to be pickled in water containing 5–10% NaCl concentration **(**Chaiyasut et al., 2018**)**. Green table olives are prepared in Arabian countries according to the Spanish style; green olive fruits are washed in 2% NaOH and then were brined at 5–10% NaCl to undergo natural lactic fermentation **(**Lamzira et al., 2005**)**.

The traditional pickles are good source of antioxidants, probiotics, vitamins (vitamin C, A, K and folate) and minerals (iron, calcium, and potassium) and considered an attractive supplement of diets **(**Chaiyasut et al., 2018**)**. Unfortunately, several harmful and pathogenic microorganisms were isolated and characterized from pickles **(**Cheng et al., 1981; Enan et al., 2018a, 2018b**)**. This clearly showed that there is a need to develop pickling ways by hygienic procedures such as using of LAB which were reported to antagonize pathogenic bacteria by their ability to produce antimicrobial proteins (bacteriocins), diacetyl, organic acids, H_2_O_2_, ethanol and acetaldehyde **(**Martinz et al., 2013; Rouhi et al., 2013; Enan et al., 2014b, Enan et al., 2014a, 2016; Reda et al., 2018**)**. The LAB metabolites were showed to inhibit the pathogenic bacteria and even inhibited the multidrug resistant variants of these bacteria **(**Enan et al. .,1996; Enan, 2000; Enan, 2006; Enan and Amri, 2006; Enan et al., 2013b, Enan et al., 2013a; Abdel-Shafi et al., 2014; Abdel-Haliem et al., 2016; Al-Mohammadi et al., 2021).

The LAB are used recently for pickling of vegetables and showed growth in pickles brine as they were showed to colonize green olive fruits and in turn absorb minerals, sugars and growth factors to their cells and to brine of pickling **(**Tassou, 1993; Sheehan et al., 2007**)**. The LAB used for pickling as starter cultures showed probiotic capability because they are associated with pickles and after ingestion they possessed many medicinal uses in gastrointestinal tract by their ability to produce lipases, cholesterol oxidase which degrade triacylglycride and cholesteryl esters, bacteriocins which inhibit the pathogenic bacteria **(**Behera et al., 2020**)**. In view of the antimicrobial compounds existed in CFS of LAB, they produce mainly organic acids which decrease the pH value to acidic levels unsuitable for the growth of bacterial pathogens (Enan et al., 2014a, 2014b, 2014c). LAB are known to produce bacteriocins (antimicrobial proteins) which are positively charged and could attach the negatively charged phospholipids of bacterial surfaces, leading to formation of pores in bacterial cell membranes; from which cell electrolytes emerge outside cells, leading to cell death (Ouda et al., 2014). Therefore, the LAB used in this work are characterized previously and produced bacteriocins and consequently are used in this study as starter cultures for pickling of green olive fruits and as an inhibitory cultures against pathogenic bacteria (Abdel-Haliem et al., 2016; Reda et al., 2018; Reda, 2019).

The present study was undertaken to inhibit some pathogenic bacteria such as *L. monocytogenes*, *B. cereus*, *S. aureus and E. coli* by three LAB namely: *L. plantarum*, *L. fermentum* and *P. acidilactici in vitro* and during making of olive pickles as these LAB strains were isolated from pickles. The effect of CFS from *L. plantarum* on both *L. monocytogenes* and *E. coli* cells was studied using TEM.

## 2 Materials and methods

### 2.1 Bacterial strains and culture media

The LAB used such as *L. plantarum*, *L. fermentum*; *P. acidilactici* were isolated from pickled green olives **(**Abdel-Haliem et al., 2016**)**; pickled green pepper **(**Reda et al., 2018**)**; mixed pickles **(**Reda, 2019**)** respectively. They were stored in glass beads in our culture collection, propagated and sub cultured into brain heart infusion broth (BHIB) (Oxoid). The three LAB strains were characterized previously and appeared to produce organic acids and antimicrobial proteins **(**Abdel-Haliem et al., 2016; Reda et al., 2018; Reda, 2019**)**.

The indicator bacteria used were Gram positive bacteria such as *B. cereus, L. monocytogenes, S. aureus* and one Gram negative bacterium such as *E. coli.* They were stored in glass beads in our culture collection, propagated and subcultured into BHIB (Oxoid).

### 2.2 Preparation of olives prior to pickling processes

Green olive fruits were harvested from the olive orchards in Belbeis Area (40 km north- Cairo). The injured fruits were removed and the right fruits were transported to the Laboratory of Food Microbiology, Faculty of Science, Zagazig University, Egypt; these fruits were put in Erlenmeyer flasks containing 2% NaOH for 6 h. Then NaOH was removed and, were washed again with sterile distilled water under completely sterilized conditions **(**Lamzira et al., 2005**)**.

### 2.3 Preparation of cell free supernatants (CFS)

The starter LAB (actively growing cells) used were grown in MRS broth for 18h as this incubation period was the optimum one for production of the inhibitory substances by these bacteria (Abdel-Shafi et al., 2014a; Enan et al., 2013a, Enan et al., 2013b; Reda et al., 2018). After the incubation period (18h), CFS preparation were prepared by centrifugation of cultures at 10000 rpm for 15 min at 4 °C; this CFS values from the starter cultures of lactic acid bacteria used were used as inhibitory agents for pathogenic bacteria during olive pickling (Enan et al., 2014a; Lamzira et al., 2005). To check whether the organic acids in CFS possess certain role in the inhibitory activity against the pathogenic bacteria used, this CFS was neutralized using 0.1 N NaOH to pH 7.0; this neutralized CFS was sterilized by filtration using membrane filters (0.45 μm, Amicon) and this neutralized and sterilized CFS designated NCFS and was used in the experiments (Enan et al., 2013a, 2013b, 2014b, 2014c, Abdel-Shafi et al., 2016).

### 2.4 Inhibition of pathogenic food-borne bacteria by either CFS or NCFS from LAB used

Inocula of actively growing cells of the indicator pathogenic bacteria of about 2.8 × 10^5^ CFU/mL were prepared and were used for inoculation of 100 mL aliquots of sterile BHI broth (Oxoid) in 250 mL Erlenmeyer flasks (Gomhuria company, Egypt that were treated with 2% of either CFS or NCFS taken from the LAB used namely: *L. plantarum*, *L. fermentum* and *P. acidilactici*. Control experiments included BHI aliquots inoculated with pathogenic bacteria and not treated with either CFS or NCFS. Samples and controls were incubated at 37 °C for 4 days. Throughout this incubation period and at appropriate time intervals, 1-mL aliquots of bacterial suspensions were withdrawn and growth values were calculated (CFU/mL) onto specific media such as Oxford *Listeria* selective agar; Baird Parker agar; Mannitol egg Yolk polymexin agar; MacConkey agar (All from Oxoid) for enumeration of *L. monocytogenes; S. aureus; B. cereus; E. coli* respectively **(**Enan, 2000; Enan et al., 2002, 2013c, 2013d, 2014c, 2015, 2018a, 2018b; Abdellatif et al., 2018**)**.

### 2.5 Inhibition of pathogenic bacteria by starter LAB during pickling of green olive fruits

The following LAB namely: *L. plantarum, L. fermentum* and *P. acidilactici* were used as starter cultures for olive pickles making and as inhibitory agents for pathogenic bacteria during pickling period (30 days). Sterile 1L glass jars with screw capped lids (Gomhuria company, Egypt) were prepared and 400mL brine were added in each jar (Sterile distilled water plus 5% NaCl); then 400g prepared green olive fruits were added. Four jars were prepared for each experiment. The first jar was control experiment that inoculated by certain pathogenic bacterium only (2 × 10^5^ CFU/mL); the second jar was inoculated by one pathogenic bacterium and *L. plantarum* (2 × 10^5^ CFU/mL); 3^rd^ jar was inoculated by one pathogenic bacterium plus *L. fermentum* (2 × 10^5^ CFU/mL); 4^th^ jar was inoculated by one pathogenic bacterium and *P. acidilactici* (2 × 10^5^ CFU/mL). There were 3 replicates for 4 experiments; each experiment included 4 jars assimilating the control and 3 jars for inhibition of 3 bacterial pathogens by either *L. plantarum* or *L. fermentum* or *P. acidilatic.* The incubation of pickling jars was carried out at 30 °C for 4 weeks. After appropriate time intervals, samples of brine (1 mL) were withdrawn and growth (CFU/mL) of the pathogenic bacteria used were calculated onto their specific media given above **(**Benkerroum, 2013; Chaiyasut et al., 2018).

### 2.6 TEM of the sensitive bacteria

Both *L. moncytogenes* (Gram positive) and *E. coli* (Gram negative) were used as target bacteria. They were grown into BHI broth incubated at 37 °C for almost 18h till obtaining actively exponentially growing cells. CFS of *L. plantarum* LPS10 was added (2% V/V) to these actively growing bacterial cells except control and further incubated at 37 °C for 6 h, then the treated bacterial cells were subjected to TEM analysis as given previously **(**Sitohy et al., 2013, 2021; Abdel-Shafi et al., 2016**)**.

Ultra-thin section was prepared for TEM. The immersion fixation of the cells was carried out as described previously **(**Abdel-Shafi et al., 2016**)**. The ultra-thin sections were examined and observed at 80 kV using a Jeol 2100 TEM at Electron Microscope unit, Faculty of Science, Zagazig University, Egypt.

## 3 Results

### 3.1 Inhibition of 3 food-borne pathogens by LAB *in vitro*

**Figure 1:**
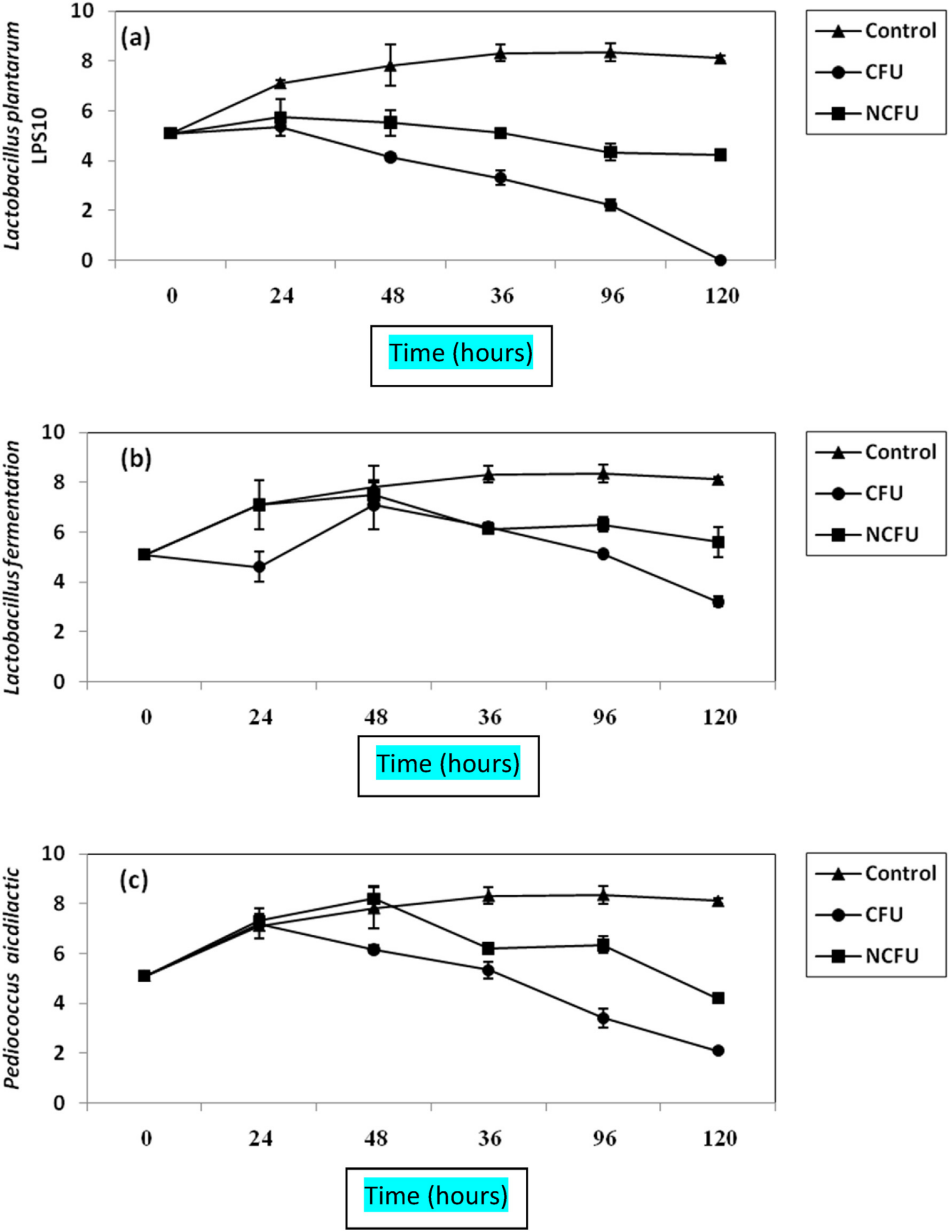
Inhibition of *L. monocytogenes* in BHI broth by both CFS and NCFS of *L. plantarum LPS10*, (a); *L. fermentum*, (b) and *P. aicdilactic,* (C). ▲; ●; ■; refer to control experiment (*L. monocytogenes* alone); *L. monocytogenes* treated by CFS; *L. monocytogenes* plus NCFS.

**Figure 2:**
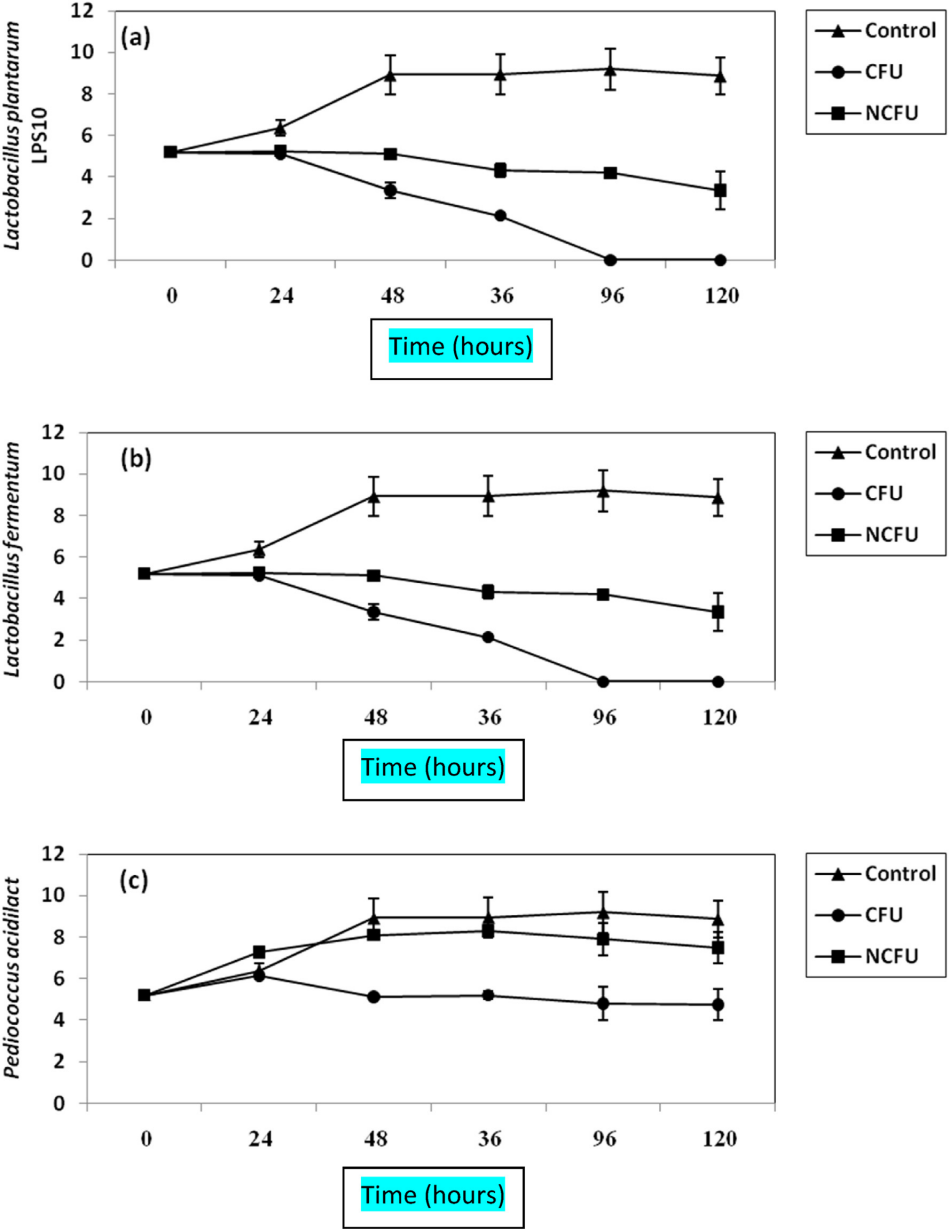
Inhibition of *S. aureus* in BHI broth by both CFS and NCFS of *L. plantarum LPS10*, (a); *L. fermentum*, (b) and *P.* aicdilactici, (C). ▲; ●; ■; refer to control experiment (*S. aureus* alone); *S. aureus* treated by CFS; *S. aureus* plus NCFS.

**Figure 3:**
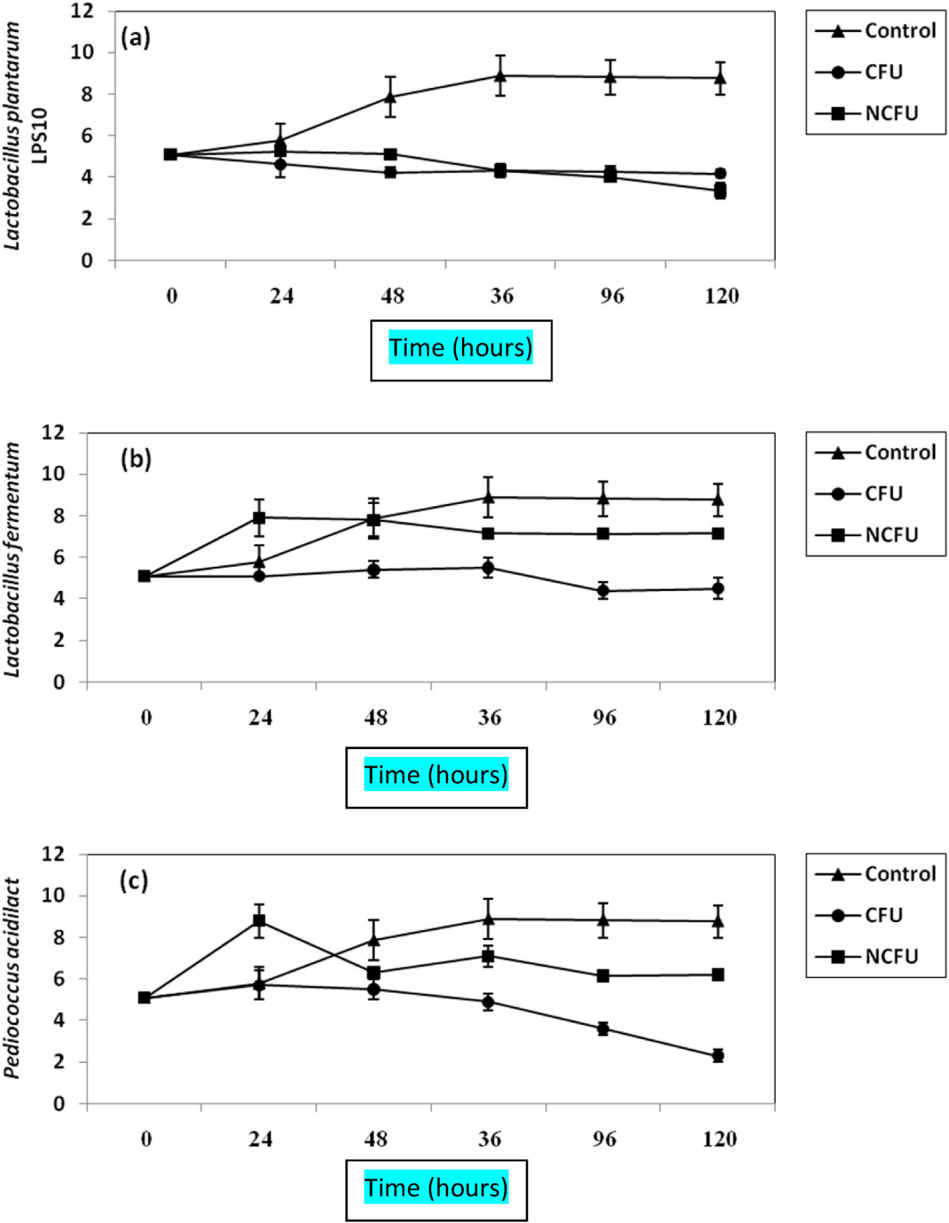
Inhibition of *B. cereus* in BHI broth by both CFS and NCFS of *L. plantarum LPS10*, (a); *L. fermentum*, (b) and *P. aicdilactic*, (C). ▲; ●; ■; refer to control experiment (*B. cereus* alone); *B. cereus* treated by CFS; *B. cereus* plus NCFS.

The inhibition of some pathogenic bacteria was studied *in vitro* (BHI broths) and in pickles by certain probiotics. Results regarding the inhibition of *L. monocytogenes* by LAB in BHI broths are given in Figure 1(a, b, c). The *L. monocytogenes* cells in control experiment (untreated samples) increased vigorously by almost 3log cycles increase within 36 h but listerious cells treated by LAB decreased distinctively (P-value ≤0.05) and difference in growth values between treated and untreated samples reached 6 log cycles, 3 log cycles; 5 log cycles after 96 h by treatments with CFS from *L. plantarum*; *L. fermentum*; *P. acidilactici* respectively [Figure 1(a, b, c)]. No growth of *L. monocytogenes* was detected after 4 days in samples treated with *L. plantarum* CFS. The samples treated with NCFS from the LAB studied inhibited slightly the listerias cells slower than that obtained by CFS [Figure 1(a, b, c)]. *S. aureus* was inhibited distinctively (P-value ≤0.05) by CFS from the LAB studied. Results are given in (Figure 2). CFS from *L. plantarum* was the more inhibitory agent than that taken from other LAB and no growth of *S. aureus* was detected after 36 h of incubation. CFS from *L. fermentum*; *P. acidlactici* inhibited distinctively *S. aureus* in BHI broths, reaching almost 6 log cycles; 4 log cycles inhibition as compared to control *S. aureus* cells (P-value ≤ 0.05) [Figure 2(a, b, c)]. In addition NCFS from the three probiotics inhibited the *S. aureus* bacterium but the inhibition was rather lower than that obtained by CFS [Figure 2(a, b, c)]. The cells of *B. cereus* in control samples (untreated samples) increased vigorously from 2.8 × 105 CFU/mL to 8.8 × 108 CFU/mL within 36 h [Figure 3(a, b, c)], however the initial growth of vegetative cells of this pathogen was declined by CFS from the three LAB studied by almost 50% after 96 h and difference in growth values between control growth and treated samples was 5 log cycles; 4 log cycles; 6 logs cycles via treatments by *L. plantarum*; *L. fermentum*; *P. acidilactici* respectively Figure [3(a, b, c)]. The NCFS from both *L. plantarum* and *P. acidilactici* inhibited also *B. cereus* vegetative cells but the inhibition was slower than that obtained by CFS; NCFS from *L. fermentum* (Figure 3).

**Figure 4:**
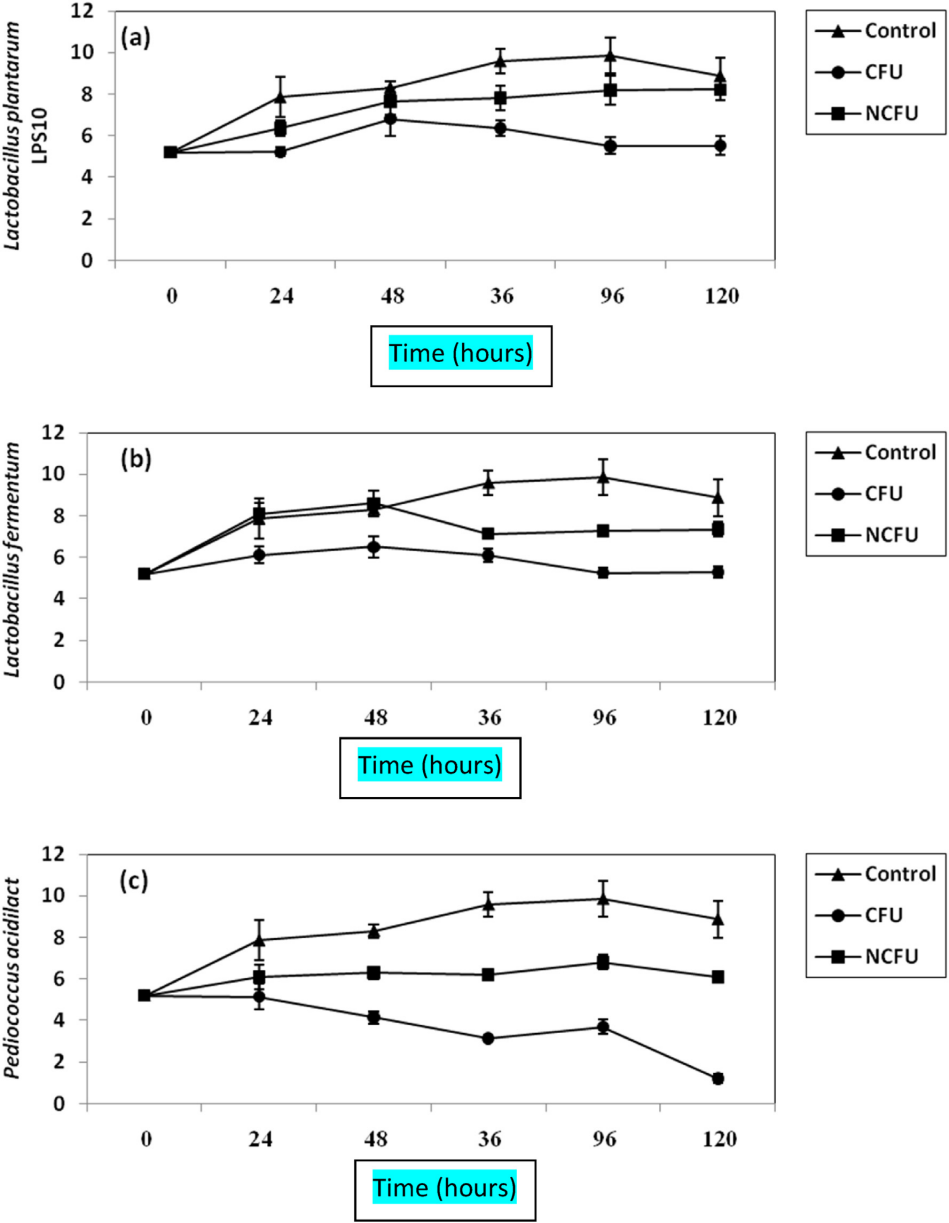
Inhibition of *E. coli* in BHI broth by both CFS and NCFS of *L. plantarum LPS10*, (a); *L. fermentum*, (b) and *P. aicdilactic,* (C). ▲; ●; ■; refer to control experiment (*E.coli* alone); *E. coli* treated by CFS; *E. coli* plus NCFS.

The effect of either CFS or NCFS from the three studied LAB on *E. coli* growth was studied. Results are given in Figure 4(a, b, c). Growth of E. coli in control (untreated samples) increased from almost 5 log CFU/mL to 9.6 log CFU/mL within 36 h, but initial inocula containing 5.2 log CFU/mL in samples treated by *L. plantarum* and *L. fermentum* continued constant and no increase or decrease of CFU/mL was detected, indicating on bacteriostatic effect [Figure 4(a, b, c)]. CFS from *P. acidilactici* inhibited *E. coli* distinctively (P-value ≤0.05) and log CFU/mL was decreased from 5.2 log CFU/mL to 1.2 log CFU/mL within 120 h (Figure 4). NCSF from the three studied LAB did not inhibit *E. coli* cells under investigation [Figure 4(a, b, c)].

### 3.2 Inhibition of food-borne pathogens by LAB during pickling of green olives

**Figure 5:**
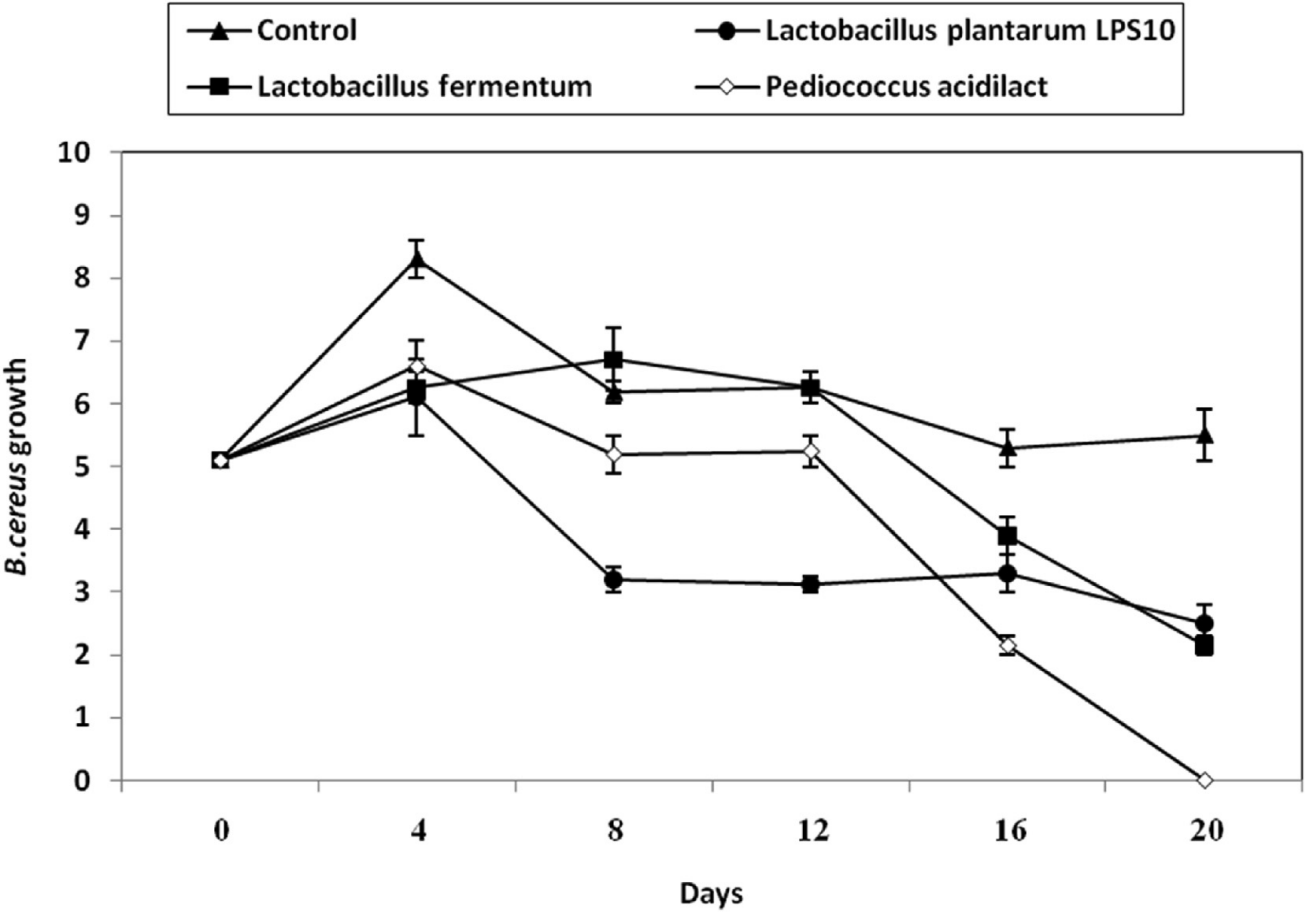
Inhibition of *L. monocytogenes* during making of olive pickles by both CFS of *L. plantarum LPS10*, (a); *L. fermentum*, (b) and *P. aicdilactic*, (C). ▲; ●; ■; X; refer to control experiment (*L.* monocytogenes cells alone), (▲); *L. monocytogenes* in pickles treated by CFS from *L. plantarum LPS10,*(●); *L. fermentum*,(■); *P. aicdilactic*,(X).

Olive pickles were made and inhibition of *L. monocytogenes* was studied during pickles marking by the LAB used. *L. monocytogenes* cells in the untreated samples (control) increased slightly by 1 log cycle increase within 4d of pickling processes (Figure 5) and almost a comparable results of *listeria* growth were detected in samples treated with the 3 LAB studied (Figure 5). By further pickling, growth of *L. monocytogenes* cells showed rapid increase, reaching almost 3 log cycles decrease of listerias growth in samples treated with the 3 studied LAB (Figure 5).

**Figure 6:**
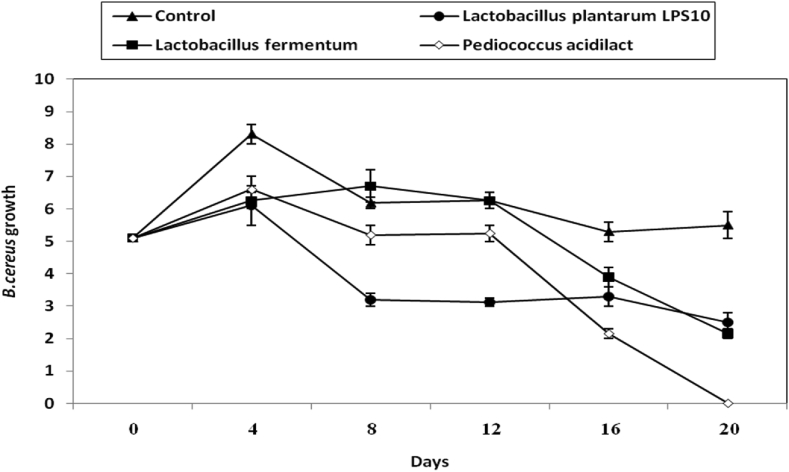
Inhibition of *B. cereus* during making of olive pickles by both CFS of *L. plantarum LPS10*, (a); *L. fermentum*, (b) and *P. aicdilactic,* (C). ▲; ●; ■; X; refer to control experiment (*B. cereus* cells alone), (▲); *B. cereus* in pickles treated by CFS from *L. plantarum LPS10*,(●); *L. fermentum*,(■); *P. aicdilactic*, (X).

The growth of *S. aureus* in the presence of the 3 experimental LAB was studied during olive pickles making. Results are given in (Figure 6). Viable cells of the three LAB declined distinctively (P-value ≤0.05) the initial growth of *S. aureus* and almost no growth of this pathogen was detected after 20 d of pickling (Figure 6).

**Figure 7:**
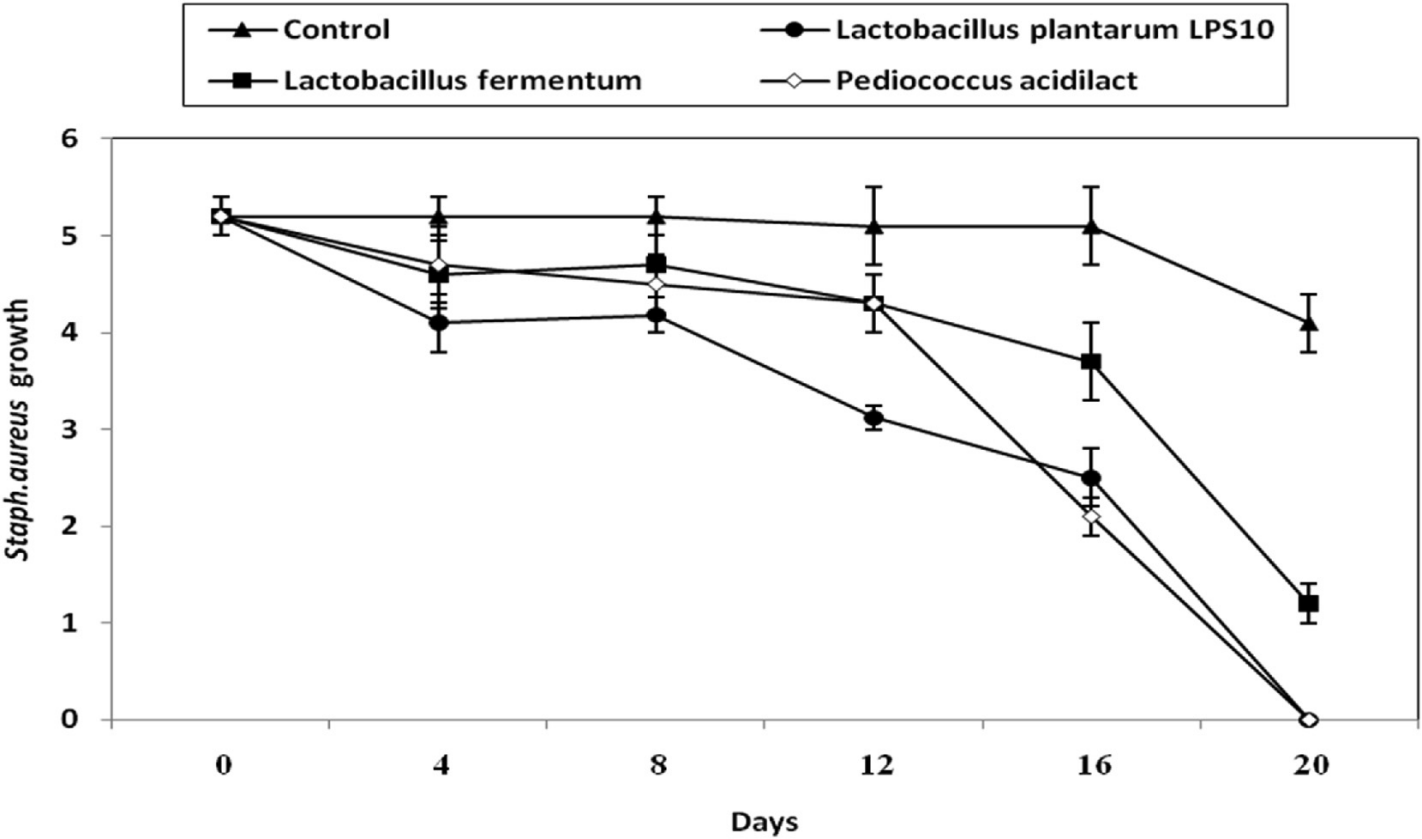
Inhibition of *S. aureus* during making of olive pickles by both CFS of *L. plantarum LPS10*, (a); *L. fermentum*, (b) and *P. aicdilactic*, (C). ▲; ●; ■; X; refer to control experiment (*S. aureus* cells alone), (▲); *S. aureus* in pickles treated by CFS from *L. plantarum LPS10*, (●); *L. fermentum*,(■); *P. aicdilactic*,(X).

The *B. cereus* vegetative cells were inhibited during olive pickles making by inoculating the pickles samples by the three LAB and 50%; 100%; 100% decline of *B. cereus* growth values were detected in olive pickling experiments treated by *L. fermentum*; *L. plantarum*; *P. acidilactici* respectively (Figure 7).

**Figure 8:**
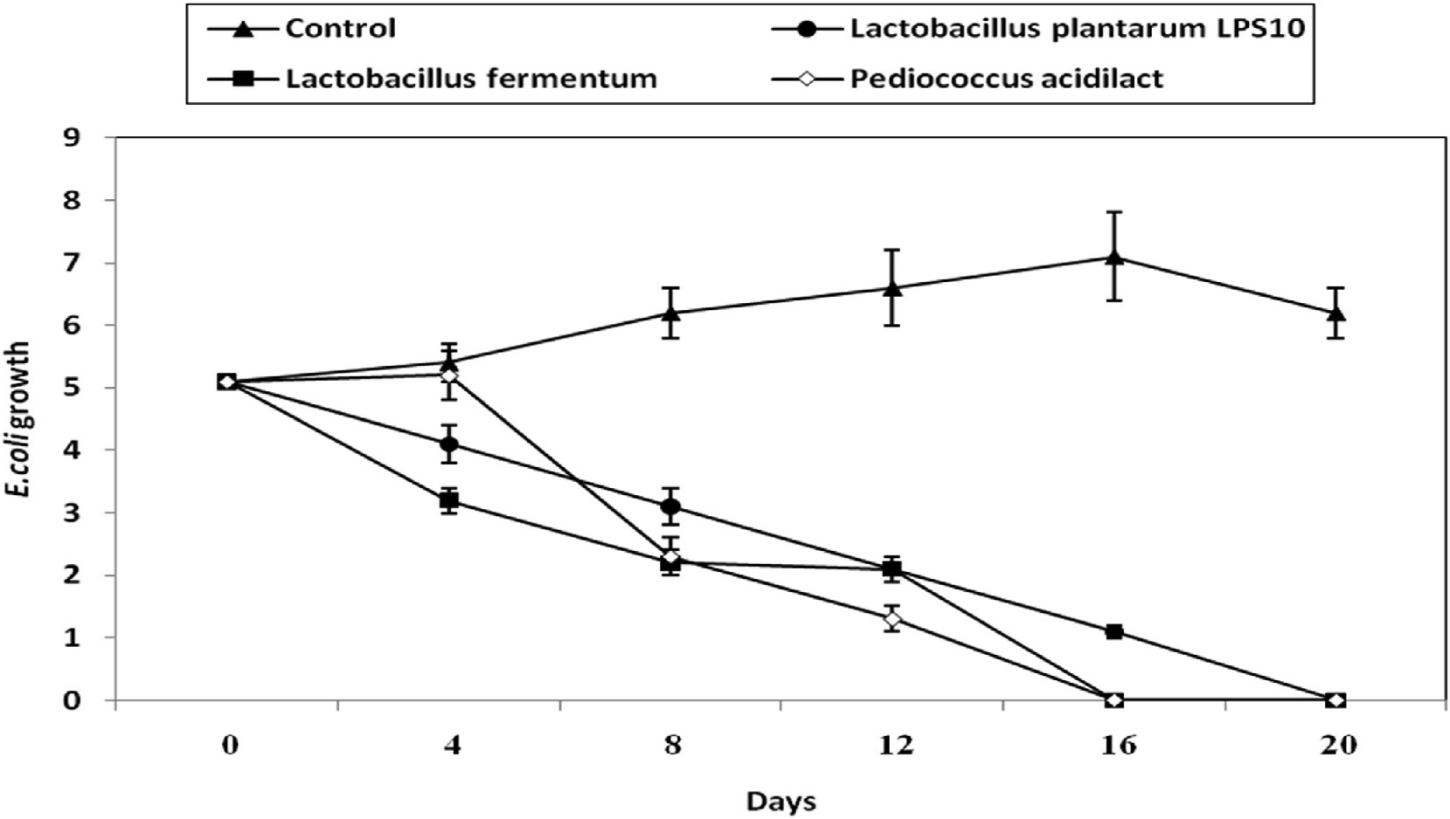
Inhibition of *E. coli* during making of olive pickles by both CFS of *L. plantarum LPS10*, (a); *L. fermentum*, (b) and *P. aicdilactic*, (C). ▲; ●; ■; X; refer to control experiment (*E.coli* cells alone), (▲); *E. coli* in pickles treated by CFS from *L. plantarum LPS10*, (●); *L. fermentum*, (■); *P. aicdilactic*,(X).

The inhibition of *E. coli* during olive pickles making was studied by cell suspension of the three studied LAB. Results are given in Figure (8). The initial inocula of *E. coli* were declined by cell suspensions of the three studied LAB and no growth of *E. coli* cells was detected almost after 16 d of incubation (Figure 8).

### 3.3 TEM examination of the CFS treated bacteria

**Figure 9:**
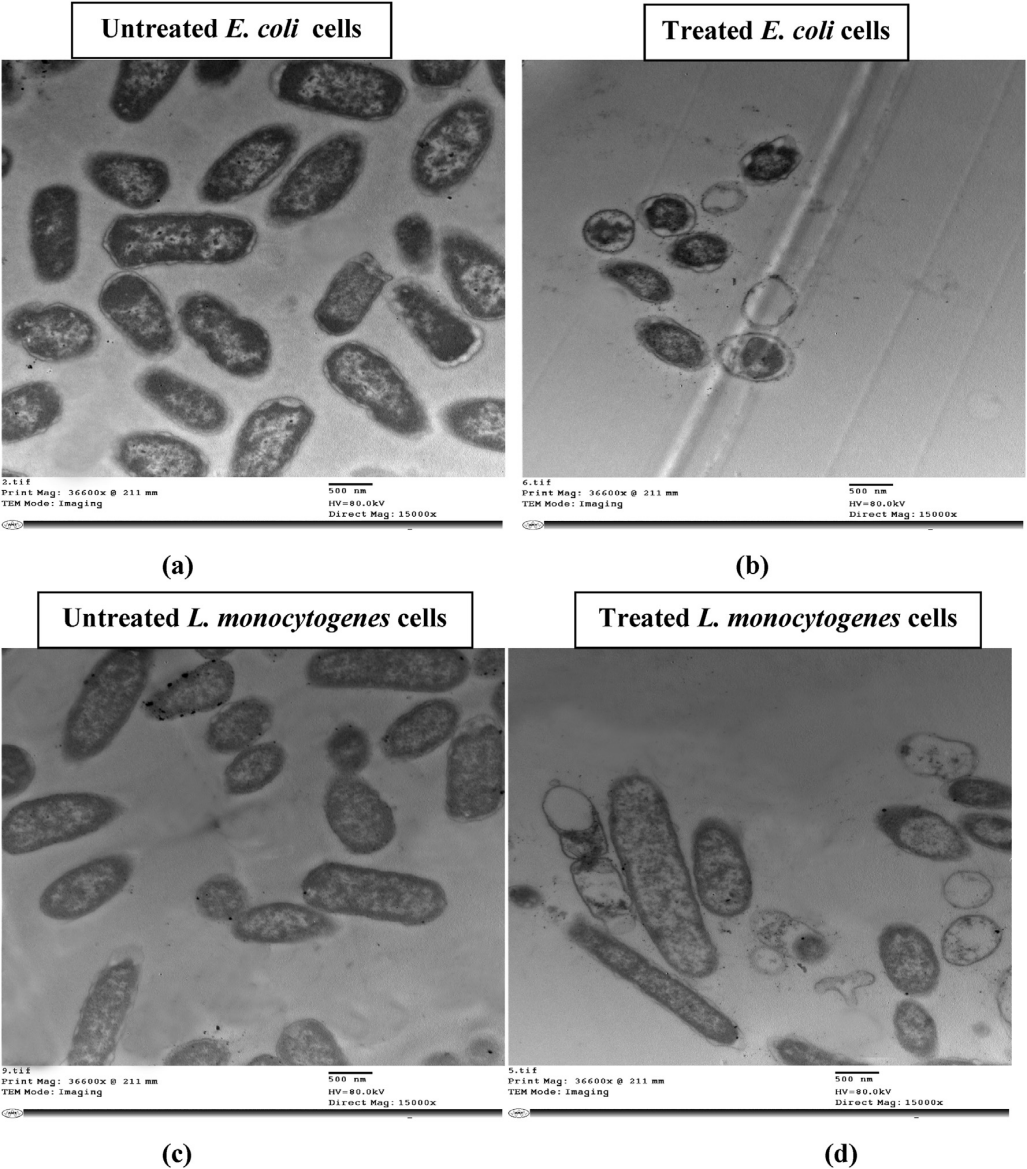
Transmission Electron microscope of bacterial cells treated by CFS from *L. plantarum* LPS10. Untreated control cells of *E. coli*, (a); treated cells of *E. coli*, (b); Untreated cells of *L. monocytogenes* (control), (c) treated cells of *L. monocytogenes*,(d).

Both *L. monocytogenes* (G + ve) and *E. coli* (G -ve) were chosen for TEM analysis to check the effect of CFS from *L. plantarum* (one example of lactic acid bacteria used) on their cells. Results are given in Figure 9(a, b, c, d). TEM image of the treated *E. coli* cells showed many cellular deformations such as clamping of cell contents and/or loss of cell contents and vacuolation of cells and cell shrinkage with disruption of cell shapes, leading certainly to cell death. The *L. monocytogenes* treated cells showed shrinkaged cells with reduced cell sizes with vacuolated spaces within cells and partially or complete loss of cell contents.

## 4 Discussion

Several studies reported the isolation of different bacterial pathogens from pickles **(**Chaiyasut et al., 2018**)**. Some of these bacterial isolates showed resistance to antibiotics **(**Sitohy et al., 2021**)**. This showed an interested challenge to develop pickling processes with complete hygienic procedures. In this regard, LAB are used as natural starter cultures for pickles making **(**Enan, 2006; Enan and Al-Amri,2006; Enan et al., 2013a, Enan et al., 2013b, 2014c**)** with inhibition of pathogenic bacteria that could grow in pickles brine by either metabolite such as organic acids, diacetyl, ethanol, acetaldehyde, carbon dioxide, antimicrobial proteins (bacteriocins). Therefore, the three lactic acid bacteria strains used herein in this study as starter cultures for olive pickling were isolated from pickles and tolerated pickles environment by their growth in pickles brine with 5% NaCl concentration **(**Abdel-Haliem et al., 2016; Reda et al., 2018; Reda, 2019**)**.

The *L. plantarum* LPS10 used in this study was isolated from pickled olives and inhibited Gram positive, Gram negative bacteria and *Candida* spp; its inhibitory activity was showed to be due to bacteriocin that designated plantaricin LPS10 which was active in the acidic pH environments (Abdel-Haliem *et al.*, 2018). *L. fermentum* PP17 was isolated from mixed pickles and showed promised starter and probiotic capabilities such as production of organic acids, growth at 6% NaCl concentration, fast growth; production of bacteriocine-like substances and tolerance to bancreatin and bile salts (2–3%) **(**Reda et al., 2018); it was, therefore, used in this study as protective and starter culture during making pickled olives. *P. acidilactici* MH512904 was isolated from olive pickles and its antimicrobial activity was due to its ability to produce organic acids and bacteriocin-like substances and H_2_O_2_ and other antimicrobial substances **(**Reda, 2019**)**. Therefore, this *P. acidilactici* MH512904 was used as starter and protective culture during making of olive pickles.

As LAB produce extracellular metabolites that contain many antimicrobial agents such as organic acids, diacetyl, acetaldehyde and bacteriocins (Enan et al., 2014a, 2014b), the CFS preparations from the three LAB tested herein were used as an inhibitory agents against the pathogenic bacteria used as indicators in this study namely: *L. monocytogenes, S. aureus B. cereus,* and *E. coli*. The CFS of the three LAB used in this study inhibited distinctively the pathogenic bacteria used as indicators namely: *L. monocytogenes, S. aureus B. cereus,* and *E. coli in vitro* and these findings support latter published results in this respect **(**Enan, 2006; Abdel-Shafi et al., 2014a; Ouda et al., 2014; Enan et al., 2014a, 2014b; Abdel-Haliem et al., 2018; Reda et al., 2018; Reda, 2019**)**. The inhibitory activity was shown previously to be due to lactic acid bacteria metabolites produced such as organic acids and bacteriocin-like substances **(**Enan et al., 2018a, 2018b; Al-Mohammadi et al., 2021**)**. CFS showed inhibitory activity against the indicator pathogenic bacteria than the neutralized CFS; this showed that organic acids existed in CFS played certain role in inhibition of the pathogenic bacteria used and such result concur with other published results in this respect **(**Enan et al., 2014a, 2014b, 2015; Abdel-Haliem et al., 2016**)**.

The three lactic acid bacteria used in this study were isolated from pickles previously **(**Abdel-Haliem et al., 2016; Reda et al., 2018; Reda, 2019**)**. Hence their growth in brine pickles were ensured and their cultures were used in this study as starter cultures for olive pickling with inhibition of the pathogenic bacteria used as indicators. Other investigations showed that lactic acid bacteria can grow in brine during pickling processes and can colonize vegetables to be pickled **(**Nychas et al., 2002**)**. Due to the colonization of LAB used as starters herein, the growth of these LAB in brine did not calculate as false results which neglect the bacterial growth colonized on pickles can be obtained. The growth of pathogenic bacteria tested in pickles brine was lower than that obtained in BHI broth, because the salt stress in pickles brine decrease the growth of these bacteria and, thus, their growth lasted long time to enable these bacterial cultures to adapt with pickles environment **(**Nychas et al., 2002; Behera et al., 2020**)**. The inhibitory activity against *L. monocytogenes, S. aureus, B. cereus* and *E. coli* in pickles could be attributed to the organic acids and antimicrobial proteins produced by LAB cultures used as starters **(**Enan et al., 1996; Reda et al., 2018; Behera et al., 2020**)**.

The analysis of TEM images for both *L. monocytogenes* and *E. coli* cells treated by CFS from *L. plantarum* showed many cellular deformations such as vacuole formation, loss of cell contents and cell shrinkage which in turn lead to cell lysis. These results are in line with latter published results in this respect **(**Enan et al., 1996; Abdel-Shafi et al., 2016; Abdel-Haliem et al., 2016**)** This showed that the inhibitory activity of CFS from *L. plantarum* was mainly due to bacteriocin and organic acids. The bacteriocin carries positively charged amino acid residues which make electrostatic forces and pore formation in cell membranes that cause leakage of cell electrolytes and in turn induce cell lysis **(**Osman et al., 2021**)**. In addition, organic acids present in CFS of *L. plantarum* were showed to be antimicrobial as they decrease pH value making acidic medium where pathogenic bacteria cannot grow (Enan et al., 2020).

Future prospective is aiming to use the lactic acid bacteria used herein for pickling of green olive fruits, for developing pickling processes of other vegetables on large scales with inhibition of other food-borne pathogens.

## 5 Conclusion

CFS from three lactic acid bacteria inhibited some food-borne pathogens *in vitro*. The cultures of those studied LAB inhibited the pathogenic bacteria used during pickling of green olive fruits. TEM examination of the CFS treated cells of both *L. monocytogenes* and *E. coli* showed cell deformations, indicating of cell lysis.

## Declaration

### Author contribution statement

Gamal Enan and Seham Abdel-Shafi: Conceived and designed the experiments; contributed reagents, materials, analysis tools or data. wrote the paper.

Eman Tartour: Performed the experiments; wrote the paper.

Abdul-Raouf Almohammadi: Conceived and designed the experiments; wrote the paper; analyzed and interpreted data.

### Funding statement

This research did not receive any specific grant from funding agencies in the public, commercial, or not-for-profit sectors.

### Data availability statement

Data will be made available on request.

### Declaration of interest's statement

The authors declare no conflict of interest.

### Additional information

No additional information is available for this paper.
